# Mitochondrial DNA analysis of eneolithic trypillians from Ukraine reveals neolithic farming genetic roots

**DOI:** 10.1371/journal.pone.0172952

**Published:** 2017-02-24

**Authors:** Alexey G. Nikitin, Inna Potekhina, Nadin Rohland, Swapan Mallick, David Reich, Malcolm Lillie

**Affiliations:** 1 Biology Department, Grand Valley State University, Allendale, Michigan, United States of America; 2 Department of Bioarchaeology, Institute of Archaeology, Ukrainian Academy of Sciences, Kyiv, Ukraine; 3 Department of Genetics, Harvard Medical School, Boston, Massachusetts, United States of America; 4 Broad Institute of Harvard and MIT, Cambridge, Massachusetts, United States of America; 5 Howard Hughes Medical Institute, Harvard Medical School, Boston, Massachusetts, United States of America; 6 School of Environmental Sciences (Geography), University of Hull, Hull, England; University of Oxford, UNITED KINGDOM

## Abstract

The agricultural revolution in Eastern Europe began in the Eneolithic with the Cucuteni-Trypillia culture complex. In Ukraine, the Trypillian culture (TC) existed for over two millennia (ca. 5,400–2,700 BCE) and left a wealth of artifacts. Yet, their burial rituals remain a mystery and to date almost nothing is known about the genetic composition of the TC population. One of the very few TC sites where human remains can be found is a cave called Verteba in western Ukraine. This report presents four partial and four complete mitochondrial genomes from nine TC individuals uncovered in the cave. The results of this analysis, combined with the data from previous reports, indicate that the Trypillian population at Verteba carried, for the most part, a typical Neolithic farmer package of mitochondrial DNA (mtDNA) lineages traced to Anatolian farmers and Neolithic farming groups of central Europe. At the same time, the find of two specimens belonging to haplogroup U8b1 at Verteba can be viewed as a connection of TC with the Upper Paleolithic European populations. At the level of mtDNA haplogroup frequencies, the TC population from Verteba demonstrates a close genetic relationship with population groups of the Funnel Beaker/ *Trichterbecker* cultural complex from central and northern Europe (ca. 3,950–2,500 BCE).

## Introduction

The Cucuteni-Trypillia culture complex dominated the cultural landscape of the Carpathian foothills in eastern Romania, Moldova and the territory of modern-day Ukraine west of the Dnieper River during the Eneolithic (Copper Age) period in eastern Europe, ca. 5,400–2,700 BCE. It is known as the Cucuteni culture in its western ranges, while in its eastern part it is known as the Trypillian culture (TC) after the village of Trypillia in what is now central Ukraine where it was first identified by Vikentij Khvoika in the late 19^th^ century [1].

Spanning more than 2,000 years, TC influenced the course of human population and cultural history in eastern Europe. Some of the best-known TC accomplishments are its proto-urban mega-sites dated to 4,100–3,600 BCE. These are architectural phenomena of communal living with each site stretching over 150 hectares with a carefully planned layout and hundreds of buildings that could house more than 10,000 people [2]. The fact that Trypillian groups carried out active trade and interactions with their neighbors is well documented in the archeological record [3]. Trypillian neighbors to the north and northwest were the Lengyel and Funnel Beaker (FBC, also *Trichterbecker* or TRB) culture groups. In the south, TC interacted with the North Pontic Region (NPR) steppe populations with which TC formed a steppe-agrarian conglomerate called Usatovo ca. 3,300 BCE, which left a lasting impression on the region and beyond. Apart from the impressive burial mounds (kurgans) the Usatovo people left behind, Usatovo also likely mediated the spread of Indo-European languages across Europe, in particular helping to forge a link between the steppe and TRB groups from southeast Poland thus facilitating the establishment of Pre-Germanic dialects [4].

The material culture of TC, including exquisitely painted pottery, an extensive array of anthropomorphic and zoomorphic clay figurines, as well as a variety of implements for land cultivating and grain processing provides strong evidence for the agrarian basis of the TC economy [3,5]. While the origin of the economic subsistence and cultural attributes of Trypillia have been traced to European Neolithic farmers, the extent of biological relatedness of the people of TC to the Neolithic European farming groups remains unclear.

The major impediment in the study of the genetic origins of the carriers of TC is that while leaving behind great volumes of material culture evidence, very little trace of TC inhabitants themselves remains. In fact, human burials are virtually unknown until the final part of TC chronology [6]. The only TC site discovered to date that contains a continuous record of human osteological deposits is a gypsum cave called Verteba located in the Podillya region of western Ukraine.

Verteba Cave (Lat: 48.47/Long: 25.53) is situated in the southwestern boreal forest-steppe zone of the East European Plain. Verteba contains the earliest human remains belonging to TC [7]. The reconstructed site chronology suggests several phases of human remains deposition, covering a substantial span of Trypillian cultural existence, ca. 3,950–2,700 BCE [7]. Human remains dated to the late Bronze Age (ca. 1,015–420 BCE) were also uncovered in the cave [8]. Thus, Verteba provides a unique record of human ritual activity spanning over 1,000 years for TC and continuing for at least another 1,500 years. The availability of human remains provides an opportunity to study the population dynamics in the area through genetic and anthropological analyses, particularly with respect to the understanding of the biological origins of TC.

An earlier archeogenetic study conducted on ancient mitochondrial DNA (mtDNA) has revealed lineages characterizing European Neolithic farmers in TC remains from Verteba [7]. No lineages identifiable as belonging to autochthonous hunter-gatherer groups were found at Verteba until the Bronze Age [8]. Anthropological studies of TC remains concurred with mtDNA data and characterized the TC population as belonging predominantly to the gracile Mediterranean type prevalent in the Neolithic farming communities of Europe and Anatolia. At the same time, the more robust craniological type characteristic of European populations of late Pleistocene as well as steppe populations of the NPR was also identified among the TC remains [9–11]. These findings established the overall conclusion about the relatedness of TC to the descendants of the Anatolian farmers and the culture itself became considered an expansion of European Neolithic farming communities eastward [3]. The presence of a robust craniological type was considered to be evidence of an admixture with local Mesolithic populations as well as populations from the North Pontic steppe [9–11], but the nature and extent of this admixture remained unclear. The small sample size and a low-resolution coverage of the early ancient DNA (aDNA) study [7] did not allow a comparable quantification of Trypillian mtDNA lineages against other prehistoric groups. In this report, seven new specimens from Verteba are presented and two previously reported samples are re-evaluated to expand our knowledge of maternal genetic determinants of TC thus making it possible to put mtDNA TC heritage in the continent-wide context of mtDNA haplogroup frequencies of prehistoric populations of Eurasia.

## Material and methods

### Origin of the specimens

The specimens reported in this study were recovered during excavations at Verteba Cave undertaken at the direction of the Borschiv Museum of Regional History and Ethnography in Borschiv, Ukraine (Mykhailo Sokhatsky, Museum and excavations Director) during the 2007–2008 field season (excavation permit # №268 / 0317, issued by the Institute of Archaeology, National Academy of Sciences of Ukraine). See [7] for a detailed description of the site excavations. The excavation finds, including human osteological material, have been deposited in a permanent repository at the Borschiv Museum of Regional History and Ethnography in Borschiv, Ukraine and are publically accessible. Samples for DNA (teeth and cranial fragments) were taken from specimens V1.1.1, V1.2, V3.13.1, V3.14.1, V3.15.1, V3.16.1, and V3.17.1. In addition, two previously reported specimens, A22 and M5 [7], were re-typed in the current study. A22 consistently produced no aDNA data in previous attempts and M5 was the better-performing specimen for aDNA in previous analyses. Based on radiocarbon dates of human remains and associated pottery sherds, specimen A22 dates to 4,000–3,400 BCE [7]. The M5 specimen was dated to 3,600–2,900 BCE [7]. The chronological age for the rest of the specimens used in the current study was placed within the range of 3,700–3,500 BCE based on radiocarbon dating of specimen V1.2 [12]). Site #7 where specimens V1.1.1-V3.17.1 have been discovered has been extensively dated using potsherds as well as human and animal remains to the range of 3,700–2,700 BCE, with the peak activity at the site around 3,500 BCE [7].

### Ancient DNA extraction and analysis–Grand Valley State University (GVSU)

Ancient DNA (aDNA) extraction and low-resolution PCR-SNP analysis of the diagnostic coding and hypervariable 1 (HVR-1) regions of mtDNA followed by Sanger DNA sequencing was performed as described in [13]. DNA extraction from specimens V1.1.1, V1.2, V3.14.1, V3.15.1, V3.16.1, and V3.17.1 was performed from single teeth in a single extraction from each tooth. DNA analysis of specimen V3.13.1 was not performed at GVSU. For M5 and A22, three independent temporary separated DNA extractions were performed on teeth, mandibular bone material (M5) and different metacarpals and phalanges (A22).

To ensure the authenticity of the results, the adherence to the best practice procedures for working with aDNA (summarized in [14]) were strictly maintained. All aDNA manipulations were conducted in a dedicated limited-access aDNA facility with two laminar flow hoods equipped with HEPA air filtration and internal UVC light systems (one hood for DNA extraction, one for PCR setup). Full body coveralls, facemasks and face shields were worn at all times during all specimen manipulation. To further reduce the chances of contamination by modern DNA through handling, all pre-PCR specimen manipulations were performed by a single person.

Bone specimens were cleaned in a separate facility under a chemical fume hood by sanding off ~1mm of the surface to remove surface contamination. Specimens were irradiated on all sides using 253.5 nm UV light. About 1g of bone was removed per extraction using a Dremel tool and powdered using a sterilized porcelain mortar and pestle. The powder was washed three times with EDTA pH 8.0 followed by three rinses with sterile water, pH 7. DNA was extracted using a QIAGEN QIAmp DNA Investigator Kit (QIAGEN Inc., Valencia, CA, USA) following a modified QIAamp protocol for extraction of DNA from bone. Negative controls were used with each extraction. Extracted DNA was eluted in 20–25 μl of sterile water and stored at -20°C.

The HVR-1 and diagnostic coding regions were amplified using previously reported primers [13]. Four primer pairs were used to amplify the HVR-1 region in overlapping segments 145–164 bp in length as described in [13]. An additional primer pair (L16185: AACCCAATCCACATCAAAACC; H16273: AGGGTGGGTAGGTTTGTTGGTATCC) producing a 133-bp fragment was used to improve the resolution at nucleotide position 16189 of HVR-1. MtDNA diagnostic coding region sites for haplogroups H (nucleotide position 7028), J/T (nucleotide position 4216) and U (nucleotide position 12308) were amplified using previously published primers [15]. Negative controls were used to detect the presence of contamination and positive controls, set up in isolation from aDNA, were used to establish effective PCR chemistry. Amplification was carried out using a QIAGEN Fast-Cycling PCR Kit as directed in the kit protocol following the conditions optimized for fragments in the 10-100-copy range. Amplification cycles were kept at 49 rounds. Each coding and control region segment was amplified up to four times per extraction or until two independent amplification products were obtained. Successful amplifications were cleaned using a Qiagen MinElute kit and eluted into 10μl of sterile water.

Successful amplifications were cloned by ligation into QIAGEN pDrive vectors using a QIAGEN PCR Cloning Kit. Transformed cells were grown on sterile LB-Amp agar plates and incubated at 37°C for 16 to 20 hours. Cells containing the PCR insert were selected by blue-white differentiation, re-plated and incubated again at 37°C for 20–26 hours. Subcultured cells were eluted into 250μL of sterile water using a sterile loop. Clone DNA amplification was performed by using 1μL of resuspended cells with SP6 and T7 universal primers to amplify the entire fragment within the plasmid cloning site. After an initial 5 minutes at 95°C to lyse cells, 29 PCR cycles were as follows: 94°C for 30 seconds, 42°C for 45 seconds, 72°C for 90 seconds with one elongation step of 72°C for 5 minutes at the end of the 29 cycles. To verify an insertion of the desired PCR fragment into the plasmid vector, PCR products were visualized on a 2.5% agarose gel.

Sanger DNA sequencing analysis was performed at the Annis Water Research Institute at GVSU. Sequencing reactions were carried out on 96-well plates using BigDye Terminator v3.1 Cycle Sequencing Kit (Applied Biosystems) for 45 rounds. Sequencing reactions were cleaned before sequencing using a standard Sephadex protocol. Samples were run on an ABI 3130x1 Genetic Analyzer with a 50-cm capillary array.

DNA sequence analysis was accomplished using the tools from NCBI BLAST (http://blast.ncbi.nlm.nih.gov/Blast.cgi) through alignment with the revised Cambridge Reference Sequence (rCRS) of mtDNA [16] (GenBank accession # NC 012920) to determine SNP differentiation. SNP variations were referenced with the phylogenetic tree of global human mtDNA variation (phylotree.org), based on both coding and control region polymorphisms, to determine haplogroup assignment. All chromatograms were thoroughly inspected using the 4Peaks DNA sequence viewer program (A. Griekspoor and Tom Groothuis, mekentosj.com) and ambiguous base assignments were manually called.

To insure the authenticity of aDNA results, multiple criteria were utilized, including DNA fragment length quantification based on Bioanalyzer data (aDNA is expected to be highly fragmented), fragment size-dependent amplification success frequency evaluation (shorter fragments should be preferentially amplified in an aDNA/ contamination mix), multiple PCR amplifications of the same region for each specimen, short length of amplified fragments (128–266 bp), overlapping fragment amplification, SNP match in overlapping fragments, and the cloning of amplified DNA fragments. The molecular behaviour of amplified fragments was examined. The sequences considered to be genuine aDNA displayed signs of deamination damage as well as having the potential for increased chimerization. Furthermore, SNP patterns from genuine aDNA were expected to make phylogenetic sense. An additional robust authenticity criterion was achieved by duplicate mtDNA analyses of the specimens at Harvard Medical School (HMS).

### DNA analysis–Harvard Medical School (HMS)

DNA extraction of the specimens listed in S1 Table for Next-Generation-Sequencing data production was undertaken at the ancient DNA laboratory at HMS following the protocol described in [17] with the modifications in [18]. After preparation of UDG-treated barcoded Illumina libraries [19] from the DNA extracts, one sample (I2109) failed amplification of the library (S1 Table). Libraries that showed successful library amplification were subjected to whole mitochondrial DNA enrichment as previously described [19] and paired end sequencing for 2x76 cycles and dual indexing was performed on an Illumina NextSeq500. We furthermore performed shotgun sequencing of all libraries. Bioinformatics analysis was performed as described in [20]. DNA analysis of specimens V3.15.1 and V3.16.1 was not performed at HMS.

### Statistical analysis

Principal Component Analysis (PCA) was carried out on mtDNA haplogroup frequencies of 37 pre-historic Eurasian populations listed in S2 Table, following the mtDNA haplogroup subdivisions identified for each population in the corresponding publication source. PCA analysis was performed using IBM SPSS Statistics software, Version 20. Population numbers and the sources of data are listed in the S2 Table. For Trypillian population from Verteba (TC), mtDNA haplogroup data for the specimens from this report were combined with the data from specimens reported in [7] substituting haplogroup identification for specimen M5 reported in [7] with more complete haplotyping information from this report. Haplotype representation from [7] is as follows: haplogroup H–two specimens (including M5), HV0 –one specimen, HV/V–two specimens, J–one specimen, T2 –one specimen.

## Results

At GVSU, all eight Verteba specimens subjected to mtDNA analysis produced ancient mtDNA sequence data (Table 1). Specimen V1.2 failed to amplify at HMS due to inhibition (S1 Table). Specimen A22 was initially typed to haplogroup H5b and produced a 17-fold mtDNA coverage at HMS, but its mtDNA contamination estimate using the contamMix software [21] suggests only 90% of sequences matching to the consensus, which is a substantial contamination rate. Specimen V1.1.1 produced 2-fold coverage at HMS with no haplogroup determination. At GVSU, specimens V3.17.1, V1.2 and A22 produced no nucleotide deviation from rCRS in the HVR-1 and the examined coding region segments. Thus, these three specimens were designated as belonging to haplogroup H. All three exhibited reduced amplification efficiency. At the same time, all three displayed deamination patterns consistent with post-mortem damage. However, based on the amplification results from A22 at HMS, and further considering that A22 failed to amplify in previous attempts at GVSU, the haplotyping results for A22 may not represent endogenous aDNA. Therefore, specimen A22 was excluded from further analysis.

**Table 1:** Single nucleotide polymorphisms (SNPs) and corresponding mitochondrial DNA haplogroups for Verteba samples from partial mtDNA sequences obtained at Grand Valley State University (GVSU) for the coding (sequence ranges: 4153–4396, 6950–7051, 12217–12308) and HVR-I (sequence range: 15974–16407) regions and complete mitochondrial genome sequences obtained at Harvard Medical School (HMS, sequence range: 1–16569). Nucleotide positions reported are changes relative to the revised Cambridge Reference Sequence (rCRS) [16].

Specimen ID, radiocarbon age (2 σ)	Mitochondrial haplogroup	SNPs (GVSU)	Number of clones sequenced (GVSU)	SNPs (HMS)
V1.1.1, 3,700–3,500 cal BCE	U8b1a2	C7028T, A12308G, T16172C, C16259T, C16266T, T16311C	32	Not determined
V1.2, 3,700–3,500 cal BCE	H	rCRS	16	Not determined
V3.13.1, 3,700–3,500 cal BCE	HV	Not determined	0	A263G, A750G, A1438G, A2706G, A4769G, C7028T, C13347T, A15326G, T16311C
V3.14.1, 3,700–3,500 cal BCE	T2b	C7028T, T16126C, C16294T, C16296T, T16304C	33	A073G, A263G, G513A, G709A, A750G, G930A, A1438G, G1888A, A4769G, A4917G, G5147A, T6293C, C7028T, G8697A, A8860G, T10463C, A11084G, A11251G, G11719A, A11812G, G13368A, A14233G, C14766T, G14905A, A15326G, C15452A, A15607G, G15928A, T16126C, C16294T, C16296T, T16304C, T16519C
V3.15.1, 3,700–3,500 cal BCE	U8b1b	C7028T, A12308G, T16189C, C16234T, T16324C	42	Not determined
V3.16.1, 3,700–3,500 cal BCE	HV0	C7028T, T16298C	16	Not determined
V3.17.1, 3,700–3,500 cal BCE	H5a	rCRS	16	A263G, C456T, A750G, T3645C, A4769G, A8860G, T14978Y, A15326G, T16304C
M5, 3,600–2,900 cal BCE	H1b	T16189Y, T16356C	30	A093G, A263G, A750G, A1438G, G3010A, A3796G, A8860G, A15326G, T16189Y, T16356C, T16519C

The HMS analysis revealed a diagnostic polymorphism in V3.17.1 for haplogroup H5a. Specimen M5, previously identified to belong to haplogroup H ([7], GenBank accession #JN098425) produced polymorphisms in both the GVSU and HMS labs that are characteristic to the H1b haplogroup. Specimen V3.13.1 was typed to haplogroup HV at HMS. Specimen V3.14.1 was typed to haplogroup T2b by both labs. Specimen V3.16.1 carried coding region and HVS-1 polymorphisms diagnostic for haplogroup HV0. The remaining two specimens, V1.1.1 and V3.15.1 have been identified as members of the U clade at GVSU. Specimen V1.1.1 failed to produce sufficient amplification at HMS (S1 Table). Specimen V3.15.1 was typed to haplogroup U8b1. Specimen V1.1.1 produced polymorphic sites consistent with its placement in haplogroup U8b1a2, due to the presence of transitions at nucleotide positions 16172 and 16259, although the diagnostic for U8b1 transitions at nucleotide positions 16189 and 16234 were not identified in V1.1.1. DNA sequences have been deposited in GenBank (http://www.ncbi.nlm.nih.gov/genbank/) under accession numbers KY198376-198382.

**Fig 1:**
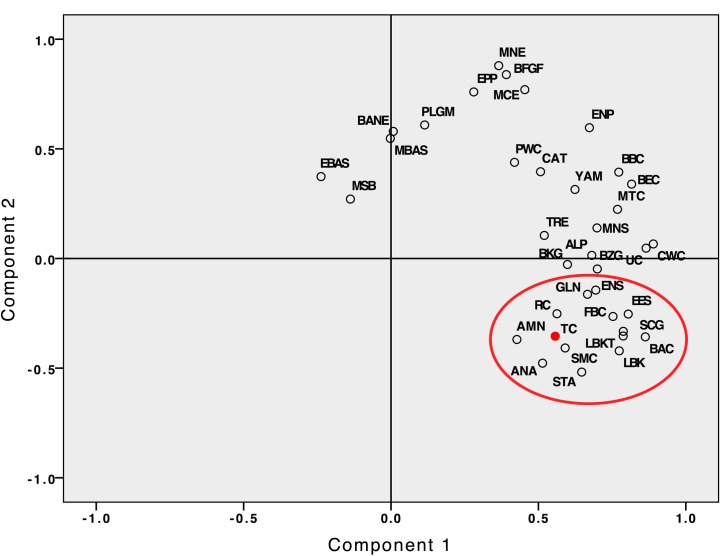
PCA plot of mitochondrial DNA frequencies of 37 Eurasian populations from the Upper Paleolithic to the Early Bronze Age, including Trypillian population from Verteba (TC, shaded). The Neo-Eneolithic cluster of farming populations from Asia Minor and Europe is circled on the graph. Culture abbreviations, population sizes and the sources of data are given in the S2 Table.

When the Trypillian mitochondrial haplogroup frequencies from Verteba from the current study were combined with the data from [7] and, along with 36 other prehistoric Eurasian populations from the Upper Paleolithic to the Bronze Age, were visualized in the space of principal components, component 1 explained 37.75% of the variance and component 2 accounted for 18.2% of the variance. On the PCA graph, the Trypillian population was placed in a cluster represented by European and West Asian/Anatolian Neo-Eneolithic farming populations (Fig 1), including Asia Minor Neolithic (AMN), Anatolian Neolithic (ANA), Neolithic from the south Paris Basin (GLN), Kriș-Starčevo from Croatia and Hungary (STA), Early Neolithic and Eneolithic Spain (ENS, EES), Linear Pottery from central Europe and Hungary (LBK, LBKT), Rossen and Schöningen from Germany (RC, SCG), as well as Funnel Beakers/TRB from Scandinavia (FBC) and Germany (Baalberge (BAC) and Salzmünde (SMC)). The Salzmünde group of German Funnel Beakers (3,400–3,025 cal BCE [22]) appeared to be the most proximate population to TC on the PCA graph.

## Discussion

The mtDNA haplogroup diversity found in the TC remains at Verteba is, overall, typical of a group of European Neolithic farmers tracing their maternal genetic roots from Anatolia with little or no admixture with indigenous hunter-gatherers. In our study, we have not identified mtDNA lineages in TC that typically characterize European Mesolithic hunter-gatherers or their Paleolithic predecessors, with a potential exception of the two U8-carrying specimens. These findings are consistent with the notion that maternal genetic contribution from Mesolithic Europeans was minimal in the TC. Thus, any robust features of craniology, if they are genetic in origin, are more likely to be explained by paternal contribution, which should be visible at the whole genome level of analysis.

The mtDNA frequency analysis presented in this report revealed close genetic association, at the mtDNA level, between TC and European Neo-Eneolithic farming groups, particularly those from central and northern Europe, including representatives of the Funnel Beaker/TRB complex such as Funnel Beakers from Scandinavia (FBC [23–27]) as well as the Baalberge (BAC) and Salzmünde (SMC) Funnel Beaker groups from central Germany (Fig 1). Like TC, the FBC group lacked representatives of hunter-gatherer lineages of haplogroup U such as U5, while the U5 component in the BAC and SMC populations comprised less than 5% of the mtDNA haplogroup variety. All three abovementioned European Funnel Beaker groups featured representatives of haplogroup H at or over 25% frequency, as well as having representatives of haplogroups J and T2b. The BAC and SMC populations also contained individuals belonging to haplogroup HV. The similarity in mtDNA lineage composition between TC and the Funnel Beaker/TRB culture complex may be a result of inter-group contacts due to the proximity of the TRB populations to the TC territory. An overlap of TC and TRB settlements has been documented to the northwest of the Verteba site, in the upper parts of the Dniester River basin and adjacent areas, and evidence of contacts between the two cultures exists in the archeological record [28]. Certain artifacts found in Verteba (clay buttons, perforated bone plates, a massive megalith inside the cave across from the cave entrance, some of these discussed in [7]) can be viewed as the influence of the Beaker cultural horizon on the Verteba cultural complex.

The geographic proximity might have promoted cultural and biological contacts between TRB and Trypillian groups throughout the entire extent of the TC distribution along the Carpathian arc and reaching the western part of the NPR, thus providing the Beakers with an access to the North Pontic steppe. A recent study revealed close genetic proximity of the Eneolithic NPR as well as western NPR Yamna groups of the Early Bronze Age to the Funnel Beaker Bernburg population (3,100–2,650 BCE) from Germany [29]. There is also evidence that the contacts involving Beakers and the Pontic steppe and forest-steppe populations potentially extended further eastward into the Ponto-Caspian region during the Early Bronze Age (EBA). An mtDNA analysis of the Novosvobodnaya and Maikop cultures (3,700–3,000 BCE) from the northern foothills of the Caucasus mountain range produced mtDNA lineages of T2b and U8b1a2 [30], although displaying different polymorphism patterns compared to the T2b and U8b1a2 lineages reported for TC (this report) and ancient farming groups from central Europe. At the same time, Novosvobodnanya culture artifacts suggest a Funnel Beaker influence [31]. Additional mtDNA sampling from Novosvobodnaya, Maikop, Trypillia and the North Pontic steppe should clarify the relationship between the EBA cultures from northern Caucasus and Trypillia and their genetic connection with the Beaker cultural horizon, as well as the extent of the Beaker influence on the genetic landscape of prehistoric Ponto-Caspian region.

Among the uncovered mtDNA lineages in the TC population at Verteba, haplogroup T2b is considered to be one of the genetic markers of the Anatolian demic expansion into Europe in the early Neolithic [22]. At the same time, ancestors of T2b may have been present in Europe since the late Pleistocene, although evidence from the studies of modern and ancient mtDNA genomes suggests that its dispersion within Europe has likely taken place in the early Neolithic period [32]. The T2b lineage is also frequent in modern populations of the Carpathian Basin. In the study of mDNA lineages of the Carpathian highlanders [33] one of the individuals coming from Bilche Zolote, three kilometers away from Verteba, carried an identical polymorphism pattern at HVS-1 to V3.14.1. Another individual from Bilche Zolote in the same study carried the H5a mtDNA lineage, thus testifying to the persistence of mitochondrial lineages derived from the European Neolithic farmers in local populations of the Verteba Cave area. At the same time, the lineage match of three of the specimens in this study (HV0 (16298), H1b and H5a) to three of the researchers (S3 Table) is most likely coincidental, at least in the latter two cases. The researcher carrying the H1b lineage did not have contact with the M5 specimen at any point in the study and the H5a lineage was determined at HMS while failing to produce an H5a-diagnostic polymorphism at GVSU.

The presence of the members of a rare in Europe U8b1 mitochondrial lineage can be viewed as further support for the link between the Eneolithic population of Verteba and Anatolian farmers. U8b1 is one of the two subclades of haplogroup U8. The other U8 subclade is haplogroup K, the dominant mitochondrial lineage in early Neolithic farmers from the Levant, Anatolia and Europe [34–37]. The U8b1b lineage identified in specimen V3.15.1 shares the HVS-1 polymorphism pattern with the Anatolian Neolithic Barcın [I0745/M11-363] specimen from [36]. In Neolithic Europe, members of the U8b1 subclade have been identified in a proto-Lengyel individual from Hungary [37] and a representative of the Schöningen group from Germany [22]. At the same time, haplogroup U8 has been reported in the Upper Paleolithic specimens from Europe [21,38]. Based on mtDNA data alone and further considering the incompleteness of the mtDNA sequence data for the U8-bearing TC specimens in this report, we cannot distinguish between the Anatolian and Paleolithic European origin of U8 lineages in TC at the present time.

Considering the data presented here and in [7], representatives of the H clade comprise 28.6% (4 out of 14 specimens) of the mitochondrial lineage composition at Verteba, which is at comparable levels with other Neo-Eneolithic European farming groups, but particularly close to the Funnel Beaker populations of Europe. The SMC Funnel Beaker group featured 30% of its lineages being members of the H clade, over half of those belonging to haplogroup H5, while the proportion of H in the BAC group was at 25% [22]. Despite the small sample size (n = 9) of available mtDNA lineages of the Scandinavian FBC group, five out of nine (55.5%) of the group’s mitochondrial lineages were represented by members of haplogroup H [23–27]. FBC and BAC also featured members of the H1 haplogroup [27]. Analyses of modern mtDNA genomes tied the origin of the main divisions of the H clade (H1, H3) to the Franco-Cantabrian region in the pre-glacial period, and their subsequent extension across Europe during the Holocene from the Iberian glacial refugium [32,39], although modifications of this scenario involving east European glacial refugia have also been proposed [33]. Some of the earliest finds of the members of the H clade in Europe, including representatives of H1 and H3, have been made in the early Neolithic sites in northeastern Spain and southern France [40–42]. Archeogenetic evidence points towards the expansion of the major sub-branches of haplogroup H such as the H1 mtDNA lineage from western Europe during the second half of the Neolithic, thus not being directly associated with the initial spread of farming in Europe, but, instead, being connected to the spread of the Beaker groups across the subcontinent [22,43]. The frequency of haplogroup H and the presence of H1 in mtDNA lineages found in TC population at Verteba further strengthen the genetic connection between TC and populations of the Beaker cultural complex.

Taken together, the maternal genetic lineages presented in this study strongly argue that the Trypillian population from Verteba derives most of its maternal genetic ancestry from the population groups that brought farming to Europe in the Neolithic. Whole genome data should further clarify the position of Trypillia on the genetic map of Eurasian prehistory.

## Supporting information

S1 Table MtDNA libraries prepared at Harvard Medical School (HMS). (DOCX)

S2 Table Cultures used in principal component and genetic distance calculations with corresponding sample sizes and references. (DOCX)

S3 Table MtDNA lineages (haplogroups) and corresponding nucleotide polymorphisms at the control (HVR-1 and HVR-2 where available) regions of archeology and anthropology personnel (AAP) as well as genetics personnel (GP) at the GVSU lab who directly handled the specimens prior to DNA analysis. (DOCX)
