# Supplementary material for: Mitochondrial DNA analysis of eneolithic trypillians from Ukraine reveals neolithic farming genetic roots
Source: PLoS One. 2017 Feb 24;12(2):e0172952. doi: 10.1371/journal.pone.0172952 (PMC5325568; doi:10.1371/journal.pone.0172952)
Supplement: S1 Table — (DOCX) [file pone.0172952.s001.docx]

| **S1 Table**. MtDNA libraries prepared at Harvard Medical School (HMS). | | | | | |  |  |  |  |
| --- | --- | --- | --- | --- | --- | --- | --- | --- | --- |
|  |  |  |  |  |  |  |  |  |  |
| **Master ID (HMS)** | **ID2** | **Specimen ID** | **mtDNA coverage** | **mtDNA haplogroup** | **mt contamination** | **mt damage in last base** | **mt damage in second to last base** | **% endogenous** | **Sex** |
| I1926 | 1V1a-H1 | 1 3.17.1 | 408 | H5a | 0.000 [2e-4-0.005] | 0.094 | 0.001 | 0.011 | M |
| I2110 | 4.V4a-H4 | 4 3.14.1 | 37 | T2b | 0.090 [0.005-0.014] | 0.106 | 0.003 | 0.0079 | M |
| I2111 | 5.V5a-H5 | 5 3.13.1 | 41 | HV | 0.028 [0.022-0.038] | 0.134 | 0.002 | 0.0033 | M |
| I1927 | 8V8a-M5 | M5 | 902 | H1b | 0.001 [3e-4-0.004] | 0.032 | 0.001 | 0.03 | M |
| I2109 | 3 V3a-H3 | 3 1.2 SE | n/a | n/a | n/a | n/a | n/a | n/a | n/a |
| I1925 | 7 V7a-H7 | 7 1.1.1 | 2 | n/a | 0.003 [5e-4-0.070] | 0.098 | 0.005 | 0.0022 | n/a |
| I1928 | 9 V9a-A22.1 | A22.1 | 1 | n/a | 0.655 [0.627-0.686] | 0.006 | 0.013 | 0.039 | n/a |
| I1929 | 9 V9a-A22.2 | A22.2 | 17 | H5b | 0.104 [0.091-0.119] | 0.114 | 0 | 0.007 | F |
