# Supplementary material for: Mitochondrial DNA analysis of eneolithic trypillians from Ukraine reveals neolithic farming genetic roots
Source: PLoS One. 2017 Feb 24;12(2):e0172952. doi: 10.1371/journal.pone.0172952 (PMC5325568; doi:10.1371/journal.pone.0172952)
Supplement: S2 Table — (DOCX) [file pone.0172952.s002.docx]

**S2 Table**. Cultures used in principal component and genetic distance calculations with corresponding sample sizes and references.

| **Abbreviation** | **Culture** | **Location** | **Sample size [ref.]** |
| --- | --- | --- | --- |
| **ALP** | Alföld | Hungary | 11 [1–3] |
| **AMN** | Asia Minor Neolithic | Syria, Turkey | 19 [4,5] |
| **ANA** | Anatolian Neolithic | Turkey | 38 [3,6–9] |
| **BAC** | Baalberge | Germany | 24 [10,11] |
| **BANE** | Bronze Age northern Europe | Russia (north) | 21 [12,13] |
| **BBC** | Bell Beaker | Germany | 48 [3,10,11,14–19] |
| **BEC** | Bernburg | Germany | 17 [10] |
| **BFGF** | Blätterhöhle | Germany | 19 [20] |
| **BKG** | Lengyel | Poland | 11 [21] |
| **BZG** | Boian-Gumelniţa | Romania | 41 [22] |
| **CAT** | Catacomb | Moldova, Russia (southwest), Ukraine (east) | 20 [23] |
| **CWC** | Corded Ware | Germany, Switzerland | 61 [3,10,18,19,24,25] |
| **EBAS** | Early Bronze Age Siberia | Siberia | 66 [26,27] |
| **EES** | Eneolithic Spain | Spain | 27 [28,29] |
| **ENP** | Eneolithic North Pontic | Bulgaria, Russia (southwest), Ukraine (south) | 16 [23] |
| **ENS** | Early Neolithic Spain | Spain | 36 [30] |
| **EPP** | Epipaleolithic Europe | Belgium, France, Germany, Italy, Spain, Switzerland | 20 [31–34] |
| **MNE** | Mesolithic northern Europe | Russia (north), Sweden | 30 [12,13,19,35–38] |
| **FBC** | Funnel Beaker/TRB | Scandinavia (Germany (north), Sweden) | 9 [38–41] |
| **GLN** | Neolithic south Paris basin | France | 55 [42] |
| **LBK** | Linear Pottery | Austria, Czech Republic, Germany, Hungary | 124 [3,10,11,15,19,36,43–46] |
| **LBKT** | Transdanubian Linear Pottery | Hungary | 39 [3,19,47] |
| **MBAS** | Middle Bronze Age Siberia | Siberia | 46 [27] |
| **MCE** | Mesolithic central Europe | Germany, Luxembourg, Poland | 17 [10,19,20,34,36,48–50] |
| **MNS** | Megalithic Spain Neolithic | Spain | 27 [51] |
| **MSB** | Mesolithic Siberia | Siberia | 52 [26] |
| **MTC** | Neolithic North Pontic | Ukraine (southeast) | 19 [52] and unpublished data |
| **PLGM** | pre-LGM Europe | Austria, Belgium, Czech Republic, Italy, France, Germany, Romania, Russia | 25 [31,32,53,54] |
| **PWC** | Pitted Ware | Sweden | 35 [10,38–40,55] |
| **RC** | Rossen | Germany | 17 [10,11,15,38,40] |
| **SCG** | Schöningen | Germany | 33 [10,11] |
| **SMC** | Salzmünde | Germany | 30 [10,11] |
| **STA** | Kriș-Starčevo | Croatia, Hungary | 46 [2,3,19,47] |
| **TC** | Trypillia | Ukraine | 14 [56], this report |
| **TRE** | Treilles | France | 29 [57] |
| **UC** | Unetice | Czech Republic, Germany, Poland | 88 [10,11] |
| **YAM** | Yamna | Bulgaria, Moldova, Kazakhstan (west), Russia (southwest), Ukraine (south) | 45 [18,19,23] |

**References**

1. Guba Z, Hadadi É, Major Á, Furka T, Juhász E, Koós J, et al. HVS-I polymorphism screening of ancient human mitochondrial DNA provides evidence for N9a discontinuity and East Asian haplogroups in the Neolithic Hungary. J Hum Genet. 2011;56: 784–796. doi:10.1038/jhg.2011.103

2. Gamba C, Jones ER, Teasdale MD, McLaughlin RL, Gonzalez-Fortes G, Mattiangeli V, et al. Genome flux and stasis in a five millennium transect of European prehistory. Nat Commun. Nature Publishing Group, a division of Macmillan Publishers Limited. All Rights Reserved.; 2014;5: 5257. doi:10.1038/ncomms6257

3. Mathieson I, Lazaridis I, Rohland N, Mallick S, Patterson N, Roodenberg SA, et al. Genome-wide patterns of selection in 230 ancient Eurasians. Nature. Nature Publishing Group; 2015;528: 499–503. doi:10.1038/nature16152

4. Fernández E, Ortiz JE, Torres T, Pérez-Pérez A, Gamba C, Tirado M, et al. Mitochondrial DNA genetic relationships at the ancient Neolithic site of Tell Halula. Forensic Sci Int Genet Suppl Ser. 2008;1: 271–273. doi:10.1016/j.fsigss.2007.10.009

5. Fernández E, Arroyo-Pardo E. Palaeogenetic study of the human remains. In: Carvalho AF, editor. Bom Santo Cave (Lisbon) and the Middle Neolithic Societies of Southern Portugal. Universidade do Algarve; 2014. pp. 133–142.

6. Hofmanová Z, Kreutzer S, Hellenthal G, Sell C, Diekmann Y, Díez-del-Molino D, et al. Early farmers from across Europe directly descended from Neolithic Aegeans. Proc Natl Acad Sci. 2016;113: 6886–6891. doi:10.1073/pnas.1523951113

7. Omrak A, Günther T, Valdiosera C, Svensson EM, Malmström H, Kiesewetter H, et al. Genomic Evidence Establishes Anatolia as the Source of the European Neolithic Gene Pool. Curr Biol. 2016;26: 270–275. doi:10.1016/j.cub.2015.12.019

8. Petrenko VG. Kurgan epokhi paleometalla na poberezh’e Khadzhubejskogo limana. MASP. 2010;11: 303–368.

9. Kılınç GM, Omrak A, Özer F, Günther T, Büyükkarakaya AM, Bıçakçı E, et al. The Demographic Development of the First Farmers in Anatolia. Curr Biol. 2016; doi:10.1016/j.cub.2016.07.057

10. Brandt G, Haak W, Adler CJ, Roth C, Szécsényi-Nagy A, Karimnia S, et al. Ancient DNA reveals key stages in the formation of central European mitochondrial genetic diversity. Science. 2013;342: 257–61. doi:10.1126/science.1241844

11. Brotherton P, Haak W, Templeton J, Brandt G, Soubrier J, Jane Adler C, et al. Neolithic mitochondrial haplogroup H genomes and the genetic origins of Europeans. Nat Commun. 2013;4: 1764. doi:10.1038/ncomms2656

12. Der Sarkissian C. Mitochondrial DNA in ancient human populations of Europe [Internet]. University of Adelaide. 2011. Available: http://hdl.handle.net/2440/74221

13. Der Sarkissian C, Balanovsky O, Brandt G, Khartanovich V, Buzhilova A, Koshel S, et al. Ancient DNA Reveals Prehistoric Gene-Flow from Siberia in the Complex Human Population History of North East Europe. PLoS Genet. 2013;9: e1003296. doi:10.1371/journal.pgen.1003296

14. Melchior L, Lynnerup N, Siegismund HR, Kivisild T, Dissing J. Genetic diversity among ancient Nordic populations. PLoS One. 2010;5. doi:10.1371/journal.pone.0011898

15. Adler CJ. Ancient DNA Studies of Human Evolution [Internet]. University of Adelaide. 2012. Available: https://digital.library.adelaide.edu.au/dspace/bitstream/2440/73014/8/02whole.pdf

16. Lee EJ, Makarewicz C, Renneberg R, Harder M, Krause-Kyora B, Müller S, et al. Emerging genetic patterns of the european neolithic: Perspectives from a late neolithic bell beaker burial site in Germany. Am J Phys Anthropol. Wiley Subscription Services, Inc., A Wiley Company; 2012;148: 571–579. doi:10.1002/ajpa.22074

17. Olivieri A, Pala M, Gandini F, Kashani BH, Perego UA, Woodward SR, et al. Mitogenomes from Two Uncommon Haplogroups Mark Late Glacial/Postglacial Expansions from the Near East and Neolithic Dispersals within Europe. Pereira LMSM, editor. PLoS One. 2013;8: e70492. doi:10.1371/journal.pone.0070492

18. Allentoft ME, Sikora M, Sjögren K-G, Rasmussen S, Rasmussen M, Stenderup J, et al. Population genomics of Bronze Age Eurasia. Nature. 2015;522: 167–172. doi:10.1038/nature14507

19. Haak W, Lazaridis I, Patterson N, Rohland N, Mallick S, Llamas B, et al. Massive migration from the steppe was a source for Indo-European languages in Europe. Nature. 2015;522: 207–11. doi:10.1038/nature14317

20. Bollongino R, Nehlich O, Richards MP, Orschiedt J, Thomas MG, Sell C, et al. 2000 Years of Parallel Societies in Stone Age Central Europe. Science (80- ). 2013;342: 479–481. doi:10.1126/science.1245049

21. Lorkiewicz W, Płoszaj T, Jędrychowska-Dańska K, Żądzińska E, Strapagiel D, Haduch E, et al. Between the Baltic and Danubian Worlds: the genetic affinities of a Middle Neolithic population from central Poland. Chaubey G, editor. PLoS One. 2015;10: e0118316. doi:10.1371/journal.pone.0118316

22. Hervella M, Rotea M, Izagirre N, Constantinescu M, Alonso S, Ioana M, et al. Ancient DNA from South-East Europe Reveals Different Events during Early and Middle Neolithic Influencing the European Genetic Heritage. PLoS One. 2015;10: e0128810. doi:10.1371/journal.pone.0128810

23. Wilde S, Timpson A, Kirsanow K, Kaiser E, Kayser M, Unterländer M, et al. Direct evidence for positive selection of skin, hair, and eye pigmentation in Europeans during the last 5,000 y. Proc Natl Acad Sci U S A. 2014;111: 4832–7. doi:10.1073/pnas.1316513111

24. Haak W, Brandt G, Jong HN d, Meyer C, Ganslmeier R, Heyd V, et al. Ancient DNA, Strontium isotopes, and osteological analyses shed light on social and kinship organization of the Later Stone Age. PNAS. 2008;105: 18226–18231. doi:10.1073/pnas.0807592105

25. Warnberg O, Alt KW. Molekulargenetische Analysen an den Bestattungen aus dem endneolithischen Kollektivgrab von Spreitenbach. In: Doppler T, editor. Spreitenbach-Moosweg (Aargau, Schweiz): ein Kollektivgrab um 2500 vChr. Bazel: Veröffentlichungder Archäologie Schweiz; 2012. pp. 158–169.

26. Weber AW, Bettinger R. Middle Holocene hunter-gatherers of Cis-Baikal, Siberia: An overview for the new century. J Anthropol Archaeol. 2010;29: 491–506. doi:10.1016/j.jaa.2010.08.002

27. Molodin VI, Pilipenko AS, Romaschenko AG, Zhuravlev AA, Trapezov RO, Chikisheva TA, et al. Human migrations in the southern region of the West Siberian Plain during the Bronze Age: Archaeological, palaeogenetic and anthropological data. Population Dynamics in Prehistory and Early History. Berlin, Boston: DE GRUYTER; 2012. doi:10.1515/9783110266306.93

28. Gómez-Sánchez D, Olalde I, Pierini F, Matas-Lalueza L, Gigli E, Lari M, et al. Mitochondrial DNA from El Mirador Cave (Atapuerca, Spain) Reveals the Heterogeneity of Chalcolithic Populations. Hofreiter M, editor. PLoS One. 2014;9: e105105. doi:10.1371/journal.pone.0105105

29. Günther T, Valdiosera C, Malmström H, Ureña I, Rodriguez-Varela R, Sverrisdóttir ÓO, et al. Ancient genomes link early farmers from Atapuerca in Spain to modern-day Basques. Proc Natl Acad Sci. 2015;112: 11917–11922. doi:10.1073/pnas.1509851112

30. Hervella M, Izagirre N, Alonso S, Fregel R, Alonso A, Cabrera VM, et al. Ancient DNA from hunter-gatherer and farmer groups from Northern Spain supports a random dispersion model for the Neolithic expansion into Europe. PLoS One. 2012;7: e34417. doi:10.1371/journal.pone.0034417

31. Fu Q, Mittnik A, Johnson PLF, Bos K, Lari M, Bollongino R, et al. A revised timescale for human evolution based on ancient mitochondrial genomes. Curr Biol. 2013;23: 553–559. doi:10.1016/j.cub.2013.02.044

32. Fu Q, Posth C, Hajdinjak M, Petr M, Mallick S, Fernandes D, et al. The genetic history of Ice Age Europe. Nature. 2016; doi:10.1038/nature17993

33. Jones ER, Gonzalez-Fortes G, Connell S, Siska V, Eriksson A, Martiniano R, et al. Upper Palaeolithic genomes reveal deep roots of modern Eurasians. Nat Commun. 2015;6: 8912. doi:10.1038/ncomms9912

34. Posth C, Renaud G, Mittnik A, Drucker DG, Rougier H, Cupillard C, et al. Pleistocene Mitochondrial Genomes Suggest a Single Major Dispersal of Non-Africans and a Late Glacial Population Turnover in Europe. Curr Biol. 2016;26: 827–833. doi:10.1016/j.cub.2016.01.037

35. Der Sarkissian C, Brotherton P, Balanovsky O, Templeton JEL, Llamas B, Soubrier J, et al. Mitochondrial genome sequencing in mesolithic North East Europe unearths a new sub-clade within the broadly distributed human haplogroup C1. PLoS One. 2014;9. doi:10.1371/journal.pone.0087612

36. Lazaridis I, Patterson N, Mittnik A, Renaud G, Mallick S, Kirsanow K, et al. Ancient human genomes suggest three ancestral populations for present-day Europeans. Nature. 2014;513: 409–13. doi:10.1038/nature13673

37. Skoglund P. Reconstructing the Human Past using Ancient and Modern Genomes. Acta Univ Ups Digit Compr Summ Uppsala Diss from Fac Sci Technol 1069. 2013; Available: http://uu.diva-portal.org/smash/get/diva2:645462/FULLTEXT01.pdf

38. Skoglund P, Malmstrom H, Omrak A, Raghavan M, Valdiosera C, Gunther T, et al. Genomic Diversity and Admixture Differs for Stone-Age Scandinavian Foragers and Farmers. Science (80- ). 2014;344: 747–750. doi:10.1126/science.1253448

39. Malmström H, Gilbert MTP, Thomas MG, Brandström M, Storå J, Molnar P, et al. Ancient DNA Reveals Lack of Continuity between Neolithic Hunter-Gatherers and Contemporary Scandinavians. Curr Biol. Elsevier; 2009;19: 1758–1762. doi:10.1016/j.cub.2009.09.017

40. Malmström H, Linderholm A, Skoglund P, Storå J, Sjödin P, Gilbert MTP, et al. Ancient mitochondrial DNA from the northern fringe of the Neolithic farming expansion in Europe sheds light on the dispersion process. Philos Trans R Soc Lond B Biol Sci. 2015;370: 20130373. doi:10.1098/rstb.2013.0373

41. Lee EJ, Renneberg R, Harder M, Krause-Kyora B, Rinne C, Müller J, et al. Collective burials among agro-pastoral societies in later Neolithic Germany: perspectives from ancient DNA. J Archaeol Sci. 2014;51: 174–180. doi:10.1016/j.jas.2012.08.037

42. Rivollat M, Mendisco F, Pemonge M-H, Safi A, Saint-Marc D, Brémond A, et al. When the Waves of European Neolithization Met: First Paleogenetic Evidence from Early Farmers in the Southern Paris Basin. Orlando L, editor. PLoS One. 2015;10: e0125521. doi:10.1371/journal.pone.0125521

43. Bramanti B. Ancient DNA: Genetic analysis of aDNA from sixteen skeletons of the Vedrovice. Anthropol (Journal Morav Zemske Muzeum). 2008;46: 153–160. Available: http://puvodni.mzm.cz/Anthropologie/article.php?ID=23

44. Haak W, Forster P, Bramanti B, Matsumura S, Brandt G, Tanzer M, et al. Ancient DNA from the first European farmers in 7500-year-old Neolithic sites. Science (80- ). 2005;310: 1016–1018. doi:10.1126/science.1118725

45. Haak W, Balanovsky O, Sanchez JJ, Koshel S, Zaporozhchenko V, Adler CJ, et al. Ancient DNA from European early neolithic farmers reveals their near eastern affinities. PLoS Biol. 2010;8: e1000536. doi:10.1371/journal.pbio.1000536

46. Zvelebil M, Pettitt P. Biosocial archaeology of the Early Neolithic: Synthetic analyses of a human skeletal population from the LBK cemetery of Vedrovice, Czech Republic. J Anthropol Archaeol. 2013;32: 313–329. doi:10.1016/j.jaa.2012.01.011

47. Szécsényi-Nagy A, Brandt G, Haak W, Keerl V, Jakucs J, Möller-Rieker S, et al. Tracing the genetic origin of Europe’s first farmers reveals insights into their social organization. Proc Biol Sci. 2015;282: 20150339. doi:10.1098/rspb.2015.0339

48. Bramanti B, Thomas MG, Haak W, Unterlaender M, Jores P, Tambets K, et al. Genetic Discontinuity Between Local Hunter-Gatherers and Central Europe’s First Farmers. Science (80- ). 2009;326: 137–140. doi:10.1126/science.1176869

49. Delsate D, Guinet J-M, Saverwyns S. De l’ocre sur le crâne mésolithique (haplogroupe U5a) de Reuland-Loschbour (Grand-Duché de Luxembourg). Bull la Société Préhistorique Luxemb. 2009;31: 7–30.

50. Sánchez-Quinto F, Schroeder H, Ramirez O, Avila-Arcos MC, Pybus M, Olalde I, et al. Genomic affinities of two 7,000-year-old Iberian hunter-gatherers. Curr Biol. 2012;22: 1494–9. doi:10.1016/j.cub.2012.06.005

51. Alt KW, Zesch S, Garrido-Pena R, Knipper C, Szécsényi-Nagy A, Roth C, et al. A community in life and death: The late neolithic megalithic tomb at Alto de Reinoso (Burgos, Spain). PLoS One. 2016;11. doi:10.1371/journal.pone.0146176

52. Nikitin AG, Newton JR, Potekhina ID. Mitochondrial haplogroup C in ancient mitochondrial DNA from Ukraine extends the presence of East Eurasian genetic lineages in Neolithic Central and Eastern Europe. J Hum Genet. 2012;57: 610–2. doi:10.1038/jhg.2012.69

53. Krause J, Briggs AW, Kircher M, Maricic T, Zwyns N, Derevianko A, et al. A Complete mtDNA Genome of an Early Modern Human from Kostenki, Russia. Curr Biol. 2010;20: 231–236. doi:10.1016/j.cub.2009.11.068

54. Fu Q, Hajdinjak M, Moldovan OT, Constantin S, Mallick S, Skoglund P, et al. An early modern human from Romania with a recent Neanderthal ancestor. Nature. 2015;524: 216–219. doi:10.1038/nature14558

55. Skoglund P, Malmström H, Raghavan M, Storå J, Hall P, Willerslev E, et al. Origins and genetic legacy of Neolithic farmers and hunter-gatherers in Europe. Science (80- ). 2012;336: 466–9. doi:10.1126/science.1216304

56. Nikitin AG, Sokhatsky MP, Kovaliukh MM, Videiko MY. Comprehensive Site Chronology and Ancient Mitochondrial DNA Analysis from Verteba Cave – a Trypillian Culture Site of Eneolithic Ukraine. Interdiscip Archaeol Nat Sci Archaeol. 2010;1: 9–18. Available: http://www.iansa.eu/papers/IANSA-2010-01-02-nikitin.html

57. Lacan M, Keyser C, Ricaut F-X, Brucato N, Duranthon F, Guilaine J, et al. Ancient DNA reveals male diffusion through the Neolithic Mediterranean route. Proc Natl Acad Sci. 2011;108: 9788–9791. doi:10.1073/pnas.1100723108
