# Supplementary material for: Mitochondrial DNA analysis of eneolithic trypillians from Ukraine reveals neolithic farming genetic roots
Source: PLoS One. 2017 Feb 24;12(2):e0172952. doi: 10.1371/journal.pone.0172952 (PMC5325568; doi:10.1371/journal.pone.0172952)
Supplement: S3 Table — (DOCX) [file pone.0172952.s003.docx]

**S3 Table**. MtDNA lineages (haplogroups) and corresponding nucleotide polymorphisms at the control (HVR-1 and HVR-2 where available) regions of archeology and anthropology personnel (AAP) as well as genetics personnel (GP) at the GVSU lab who directly handled the specimens prior to DNA analysis.

| **Individual** | **Hg** | **16189** | **16294** | **16296** | **16298** | **16304** | **16356** | **263** |
| --- | --- | --- | --- | --- | --- | --- | --- | --- |
| rCRS |  | T | C | C | T | T | T | A |
| AAP1 | H1b | C | . | . | . | . | C | G |
| AAP2 | HV0 | . | . | . | C | . | . |  |
| GP | H5a | . | . | . | . | C | . |  |
